# Sensorineural Hearing Loss and Systemic Autoimmune Disease: The Experience of a Systemic Immune-Mediated Diseases Unit

**DOI:** 10.7759/cureus.14075

**Published:** 2021-03-24

**Authors:** Renata Ribeiro, João F Serôdio, Marta C Amaral, Joana A Duarte, Carolina Durão, Nuno Mendes, José Delgado Alves

**Affiliations:** 1 Systemic Immune-Mediated Diseases Unit, Department of Internal Medicine IV, Hospital Prof. Doutor Fernando Fonseca, Amadora, PRT; 2 Immune Response and Vascular Disease, Centro de Estudos de Doenças Crónicas - CEDOC, Chronic Diseases Research Center, NOVA Medical School, Lisboa, PRT; 3 Department of Otorhinolaryngology, Hospital Prof. Doutor Fernando Fonseca, Amadora, PRT

**Keywords:** sensorineural hearing loss, autoimmune inner ear disease, systemic autoimmune disease

## Abstract

Background

Autoimmune inner ear disease (AIED) represents less than 1% of all cases of sensorineural hearing loss (SNHL) but its frequency may be underestimated due to lack of specific clinical and laboratory criteria. AIED can be associated with a systemic autoimmune disease (SAID) in 15%-30% of the cases. The objective of the present study was to characterize the clinical and prognostic factors of a cohort of patients with AIED.

Materials and methods

The authors conducted a retrospective descriptive analysis of a cohort of patients with AIED referred from the otorhinolaryngology department to a systemic immune-mediated diseases unit between March 2013 and November 2020. A consecutive sample of 39 patients with suspected AIED was referred. SNHL was defined as a fall of the hearing threshold of at least 30 decibels in three consecutive frequencies. Eight patients were excluded for not meeting the audiometric criteria or having confounding factors. The remaining 31 patients were included with a total of 50 affected ears. To classify the intensity of hearing loss, an arithmetic mean of pure tone was calculated. Normal hearing or mild hearing loss at the last pure tone audiometry of the follow-up were an indicator of good prognosis and were considered the outcome of interest.

Results

Thirty-two percent of the patients had an associated SAID. There were no differences regarding demographic and clinical characteristics when comparing patients with AIED alone and patients with AIED and a SAID, except for the positivity of antinuclear antibodies (ANA). ANA positivity was superior in patients with the association of AIED and a SAID when compared with patients with AIED alone (90% vs 50%; p=0.037). The SAID was diagnosed after the AIED in 70% of the patients, in which diagnosis of the SAID occurred a median of 4,2 (IQR 0.8-5.1) years after the diagnosis of the AIED. Normal audiometric evaluation or a mild hearing loss was achieved in 31% of the ears at the last audiometric evaluation. A normal audiometry or a mild hearing loss at the time of diagnosis was independently associated with a better outcome (31%, 14%, CI 1.71-273.69; p=0.018). Bilateral hearing loss was independently associated with a worse outcome (54%, 79%, CI 0.01-0.84; p=0.035). The use of systemic corticosteroids (p=0.941), transtympanic corticosteroids (p=0.700) and non-steroid immunomodulator drugs (p=0.986) did not affect prognosis. The presence of a SIAD did not affect the prognosis (p=0.986).

Conclusions

In this cohort, SAID was present in one-third of the patients with AIED. A good prognosis was achieved in one-third of the patients. A normal audiometry or mild disease at presentation was associated with a good outcome, whilst bilateral involvement was associated with a bad one. Association of a SAID did not seem to influence the hearing-related prognosis. Positivity of ANA antibodies may justify performing a complementary investigation to determine the presence of a SAID.

## Introduction

Sensorineural hearing loss (SNHL) has been defined as a fall of at least 30 dB of the hearing threshold in at least three consecutive frequencies [1]. It has an incidence that varies from five to 20 individuals per 100,000 per year [1,2]. Although in 70% to 90% of the cases the etiology is not identified, vascular injury, rupture of membranes, bacterial or viral infection, and immune-mediated injuries have been proposed as mechanisms of disease [1]. Autoimmune inner ear disease (AIED) represents less than 1% of all cases of SNHL [1,3], but may be underestimated due to a lack of specific clinical and laboratory criteria [1,2]. It is more common in female patients between the third and sixth decades of life [1,3].

AIED usually presents as a subacute, rapidly progressive, often fluctuating, bilateral and asymmetrical SNHL [1-4]. Almost 80% of the patients have involvement of both ears [2,5]. In the early stages of disease hearing loss may be unilateral with the contralateral ear being affected days to years after presentation [1,3]. Vestibular symptoms such as imbalance, ataxia and vertigo, may accompany the hearing loss in 50% of AIED patients [3]. Tinnitus and ear fullness are present in 25% to 50% of cases, so, in the absence of trauma and signs of infection, differential diagnosis with Meniere’s disease has to be considered [2,3,5].

The pathophysiology of AIED is not entirely understood. The concept of the inner ear as an immune-privileged site has been refuted [2]. Antibody-antigen reactions with autoantibody development (type II immune responses), activation of the complement system, deposition of immune complexes (type III immune responses), direct damage mediated by cytotoxic T-cells crossing the blood-labyrinthine barrier and reaching the endolymphatic sac, vasculitis, micro-thrombosis and electrochemical reactions have all been proposed as mechanisms of vestibular and cochlear damage [1,3,4,6]. The recruitment of immunocompetent cells and the promotion of an adaptative immune response occurs following the presence of immune mediators such as IL-1b, IL2 and TNFa [2,4]. Studies have suggested an association between SNHL and vestibulocochlear antibodies against antigens present in the inner ear such as heat shock protein 70 (HSP70), cochlin, B tectorin and type II and type IX collagen [4,5].

Diagnosis is based on the clinical presentation as there are no diagnostic criteria or pathognomonic laboratory tests. Essentially, AIED is a diagnosis of exclusion, suspected in case of a documented progressive SNHL, when other etiologic causes have been ruled out. Meniere’s disease should be considered in the presence of vestibular symptoms and retro-cochlear pathology should be excluded by computed tomography or magnetic resonance imaging [3].

AIED can be classified as primary, when the inner ear is the only organ affected, or secondary when, in 15-30% of cases, it develops in association with a systemic autoimmune disease (SAID) such as systemic lupus erythematosus (SLE), rheumatoid arthritis (RA), granulomatosis with polyangiitis, Vogt-Koyanagi-Harada disease or antiphospholipid syndrome (APS) [3,4].

The recommended treatment is systemic corticosteroids, with transtympanic infusion being preferred in cases of failure, incomplete response or contraindication to systemic therapy [1]. However, only a small percentage of patients is steroid-responsive and even in those cases, clinical response to treatment is present usually only in the first days or weeks of disease [3]. Non-steroid immunosuppressant drugs are usually used as an alternative in non-responders, as a steroid-sparing agent in responders or when indicated for a systemic associated disease [1]. Plasmapheresis has been reported in severe and steroid-resistant cases. After failing to prevent disease progression, cochlear implantation might be a strategy to replace function in patients with profound bilateral SNHL [2].

## Materials and methods

The authors conducted a retrospective descriptive analysis of a cohort of patients with SHNL referred from the otorhinolaryngology department to a systemic immune-mediated diseases unit between March 2013 and November 2020 for a suspected AIED. Diagnosis of SNHL was based on clinical presentation and the presence of a fall of the hearing threshold of at least 30 decibels (dB) in three consecutive frequencies. The diagnosis of associated SAID was applied when appropriated and was based on clinical and serologic criteria according to the respective EULAR recommendations. 

Data were collected from hospital records and handled in an anonymous and population-based fashion. Patient’s audiometric evaluations were performed with a standard audiometer (Grason-Stadler, Inc, Eden Prairie, MN). To classify the intensity of hearing loss, an arithmetic mean of pure tone was calculated. The arithmetic mean of frequencies 250 Hz, 500 Hz, 1 KHz and 2 KHz was obtained when low and medium frequencies were affected; 1, 2, 3, 4, 6 and 8 KHz when medium and high frequencies were affected; 3, 4, 6 and 8 KHz when only high frequencies were affected and of all eight frequencies when low, medium and high frequencies were affected together. In cases of deep loss, when the hearing threshold was not detected, the maximum response audiometer of 120 dB was considered. The intensity of hearing loss was classified as mild when the arithmetic mean of pure tone was between 26 and 40 dB, moderate when between 41 and 70 dB, severe if between 71 and 90 dB and profound if above 90 dB. The first and last pure tone audiometry available were used. In cases of bilateral involvement but with only one ear affected at first presentation, the contralateral one was considered normal. Anti-HSP70 antibodies were determined by an immunoenzymatic assay. Antinuclear antibodies (ANA) determination was performed by indirect immunofluorescence followed by immunoblot. The definition of positivity was expressed as a titre of at least 1:160. Anti-neutrophil cytoplasmic antibodies (ANCA) were determined by indirect immunofluorescence followed by enzyme-linked immunosorbent assay (ELISA).

Categorical data were presented as counts and percentages and were analyzed with chi-square test and Fisher’s exact test, as appropriate. Normality was tested with Kolmogorov-Smirnov test. The skewed distributions were described with medians and interquartile ranges (IQR) and were compared by the Mann-Whitney. Normal distributions were described with means and standard deviations and were compared with the use of student’s t-test. For the comparison of more than two variable groups chi-square test or ANOVA analysis were used accordingly. For the analysis of prognosis, an analysis of each ear affected by AIED was performed. In order to assess prognostic predictors, variables were selected for a logistic regression analysis, assessed by the estimated odds ratio (OR) with 95% confidence interval (CI). Variables yielding p values of less than or equal to 0.20 in the univariate analyses were entered into a multiple logistic regression model. Normal hearing or mild hearing loss at the last pure tone audiometry of the follow-up were an indicator of good prognosis and were considered the outcome of interest. The two-sided alpha level was set at 0.05.

## Results

Population

Between March 2013 and November 2020, 39 patients with suspected AIED were referred to the systemic immune-mediated diseases unit. Of these, eight were excluded. Three had vestibular symptoms and hearing loss complaints without meeting audiometric criteria for SNHL. Five were considered to have confounding factors for the purpose of the present study: one had history of otosclerosis and used bilateral hearing aids; one had a history of chronic otitis media; one had had labyrinthitis following trans-tympanic steroid infusion; one suffered from hearing impairment since childhood; one had an erratic follow-up with presentation, evolution and therapy difficult to understand from hospital records. The remaining 31 patients fulfilled the criteria for this study.

Patients’ characteristics are summarized in Table 1. Included patients were followed-up for a median of 29 (8-86) months, corresponding to 117,1 patient-years. The median age at the time of diagnosis was 51 (37-61) years and there was a clear female predominance (20 patients, 65%). In 19 patients (61%) the disease was bilateral. Either vestibular symptoms or tinnitus were present in 77% of patients (n=24). In 27 patients, anti-HSP70 was assessed being positive in 15 (56%). Sixteen patients (52%) received at least one transtympanic infusion of corticosteroids. Systemic corticosteroid treatment was used in 23 (74%) patients. Ten patients (32%) received non-steroid immunosuppressant drugs, most often in relation to a systemic autoimmune disease. One patient was treated with a cochlear implant.

**Table 1:** Comparison of clinical, immune and treatment features between idiopathic autoimmune inner ear disease and autoimmune inner ear disease in association with systemic autoimmune disease. *Vestibular symptoms or tinnitus. AIED: autoimmune inner ear disease; NA: not applicable; SAID: systemic autoimmune inner ear disease; IQR: interquartile range.

	All patients (n=31)	AIED alone (n=21)	AIED with SAID (n=10)	p-value
Age at diagnosis, years (IQR)	51 (37-61)	55 (39-62)	51 (32-58)	0.499
Female sex (%)	20 (65%)	12 (57%)	8 (80%)	0.202
Time from symptoms - diagnosis, days (IQR)	3 (0-152)	2 (0-61)	67 (2-315)	0.152
Bilateral deafness (%)	19 (61%)	12 (57%)	7 (70%)	0.389
Accompanying symptoms* (%)	24 (77%)	17 (81%)	7 (70%)	0.401
Anti-HSP70 (%)	15/27 (56%)	12/19 (63%)	3/8 (38%)	0.212
ANA (%)	19/30 (63%)	10/20 (50%)	9/10 (90%)	0.037
Follow-up, months (IQR)	29 (8-86)	21 (7-37)	85 (71-91)	0.004
Transtympanic corticosteroids (%)	16 (52%)	12 (57%)	4 (40%)	0.306
Systemic corticosteroids (%)	23 (74%)	15 (71%)	8 (80%)	0.483
Immunomodulator treatment (%)	10 (32%)	1 (5%)	9 (90%)	0.001
Cochlear implant (%)	1	0	1	NA

Systemic autoimmune disease and autoimmune inner ear disease

Ten patients (32%) with AIED also had a SAID. Four patients had SLE, three had systemic sclerosis (SS), two RA and one APS. In two patients the diagnosis of SAID occurred four and 20 months before hearing loss, respectively. In one patient the diagnosis was simultaneous. In seven patients (70%) the diagnosis of the SAID occurred a median of 4.2 (0.8-5.1) years after the diagnosis of AIED. 

When compared with patients with AIED alone, patients with the association of AIED and SAID had similar age and sex predominance (Table 1). There were also no differences regarding the presentation of deafness, laterality or the occurrence of accompanying symptoms. The prevalence of anti-HSP70 among these patients was 38% (3/8), compared to 63% (12/19) in patients with AIED alone (p=0.212). Positivity of ANA was superior in patients with the association of AIED and SAID (90% vs 50%, p=0.037). No patient was found to have positive ANCA antibodies. The proportion of patients receiving transtympanic (AIED+SAID 40% vs AIED only 57%, p=0.306) and systemic corticosteroids (AIED+SAID 80% vs. AIED only 71%, p=0.483) was similar in both groups. Cumulative systemic corticosteroid doses were also no differences between groups (p=0.152). Non-steroid Immunomodulators were used more frequently in patients with associated SAID (AIED+SAID 90% vs AIED only 5%, p=0.001). Patients with a SAID also had a longer follow-up time. The hearing-associated prognosis was similar between both groups (Table 2, p=686).

Prognosis and predictors of good outcome

The intensity of hearing loss in the final audiometric evaluation during follow-up was classified as mild, moderate, severe or profound according to the arithmetic mean of the pure tones obtained. Good outcome was considered when the final audiometry was normal or the intensity of hearing loss was mild. A total of 50 ears were affected. At the time of the analysis the final audiometry was unavailable in three patients with bilateral affection. Therefore, the final analysis included 42 ears.

**Figure 1:**
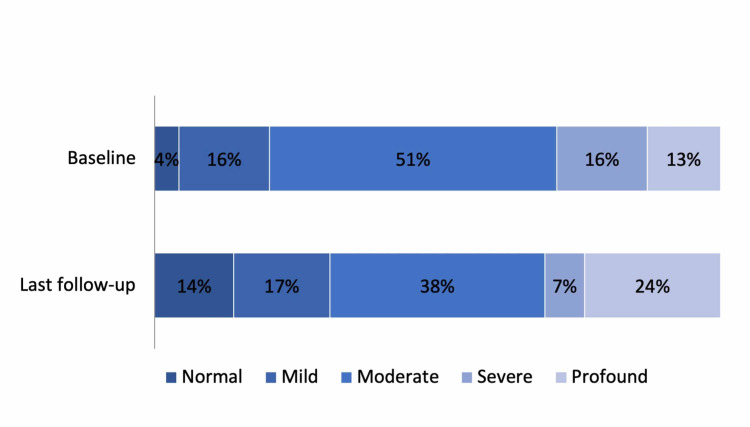
The evolution of the audiometric evaluations between the first and the last audiometric assessment during follow-up.

**Figure 2:**
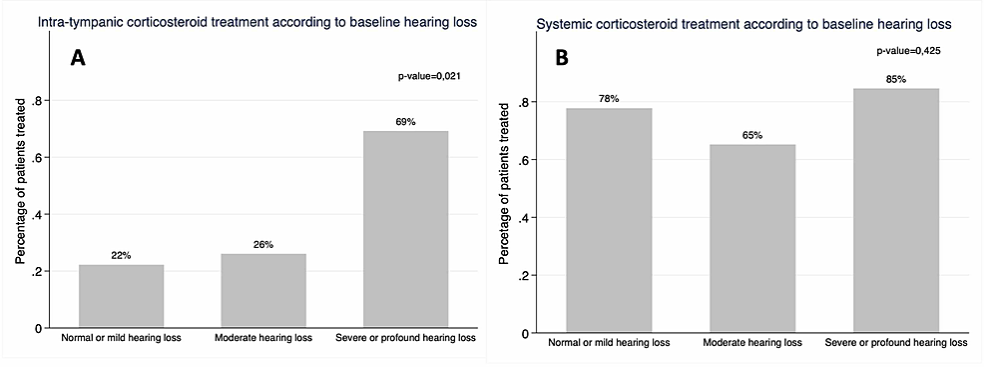
Transtympanic and systemic corticosteroid treatment according to the baseline severity of hearing loss. A shows that a higher proportion of patients with severe-profound hearing loss received transtympanic corticosteroids (p=0.021). B shows there was no difference concerning the use of systemic corticosteroids (p=0.425).

At the time of presentation, 20% of the ears had normal audiometry or a mild hearing loss, 51% had moderate and the remainder 29% had severe or profound hearing loss. At the time of the last evaluation, 31% of the ears had a normal audiometric evaluation or a mild hearing loss, 38% had moderate and 31% had severe or profound hearing loss (Figure 1). Roughly, 29% registered worsening of hearing loss during follow-up, 45% remained stable and 26% of the ears improved. Ears with severe or profound hearing loss at baseline received more frequently transtympanic corticosteroids (p=0,021). There was no difference in the proportion of ears receiving systemic steroids (p=0.425), neither in the cumulative dose of systemic corticosteroids (p=0.343) (Figure 2).

Table 2 presents the variables studied in the logistic regression model that evaluated predictors of prognosis. A normal audiometry or a mild hearing loss at the time of diagnosis was independently associated with a better outcome (p=0.018). Bilateral hearing loss (p=0,035) was independently associated with a worse prognosis. Older age also tended to associate with worse prognosis (p=0.069). The use of systemic corticosteroids (p=0.941), transtympanic corticosteroids (p=0.700) and non-steroid immunomodulator drugs (p=0.986) did not affect prognosis as did not the cumulative dose of systemic corticosteroids (p=0,344).

**Table 2:** Univariate and multivariate regression model of prognosis for all ears affected. Good outcome considered as normal audiometry or mild hearing loss at the last pure tone audiometry performed in the follow-up. *Vestibular symptoms or tinnitus. ANA: antinuclear antibodies; IQR: interquartile range.

Characteristics	Univariate analysis			Multivariate analysis	
	Good prognosis (n=13)	Bad prognosis (n=29)	p-value	Odds ratio (95% confidence interval)	p-value
Age at diagnosis, years (IQR)	39 (37-58)	55 (46-62)	0.041	0.93 (0.87-1.01)	0.069
Female sex (%)	10 (77%)	15 (52%)	0.133	1.74 (0.23-13.00)	0.589
Time from symptoms - diagnosis, days (IQR)	2 (2-78)	3 (1-61)	0.552		
Normal or mild hearing loss at diagnosis (%)	4 (31%)	4 (14%)	0.090	21.60 (1.71-273.69)	0.018
Severe or profound hearing loss at diagnosis (%)	2 (15%)	10 (34%)	0.399		
Sudden deafness (%)	10 (77%)	18 (62%)	0.350		
Bilateral deafness (%)	7 (54%)	23 (79%)	0.099	0.09 (0.01-0.84)	0.035
Accompanying symptoms* (%)	10 (77%)	22 (76%)	0.941		
Systemic autoimmune disease	4 (31%)	9 (31%)	0.986		
Anti-HSP70 (%)	6 (46%)	17 (59%)	0.794		
ANA (%)	9 (69%)	20 (69%)	0.700		
Transtympanic corticosteroids (%)	5 (38%)	13 (45%)	0.700		
Systemic corticosteroids (%)	10 (77%)	22 (76%)	0.941		
Immunomodulator treatment (%)	4 (31%)	9 (31%)	0.986		

## Discussion

Despite an increase in clinical awareness and in the knowledge of the immune mechanisms involved, AIED is still a challenging diagnosis as it is necessary to conjugate the presence of SNHL with data related to the clinical presentation, audiological testing and response to immunomodulatory drugs [7].

The authors performed a descriptive analysis of a cohort of patients with suspected AIED referred from an otorhinolaryngology department to a systemic immune-mediated diseases unit and evaluated the characteristics of the patients with associated SAID. The cohort of 31 patients with SNHL presented is mostly in accordance with the reports available. Patients are most frequently female, have the diagnosis made between their fourth and seventh decade of life and disease is usually bilateral. There is a common belief that AIED, specifically when associated with a SAID, is a progressive disease that involves both ears and many studies included only cases with bilateral hearing loss [8]. However, given that the clinical presentation of AIED can be sudden and unilateral [8], sometimes taking years to become bilateral [7], the authors chose to include patients with unilateral and bilateral hearing loss. 

Nearly one-third of the patients had an associated SAID, in the majority of the cases diagnosed after de diagnosis of the SNHL. There were no differences in patient or disease-related features at presentation when comparing patients with and without associated SAID, except for the presence of ANA. In the cohort presented, the most frequently associated SAID was SLE, a systemic disease where the most common form of otologic involvement is SNHL, with a reported prevalence of six to 70% [9]. Mechanisms such as humoral responses with antibody-antigen reactions, cell-mediated cytotoxic damage to cochlear and vestibular organs, and immunocomplexes deposition in microvessels have been proposed, probably in association with micro-thrombosis when APS is present [9]. SS and RA have associated SNHL reported in 20% to 27% [10,11] and 25 to 72% [12] of cases, respectively. Auditory neuropathy due to vasculitis has been proposed as an additional cause for SNHL in RA [13]. In its turn, APS is a state of hypercoagulability which might induce micro-thrombosis with the subsequent clinical consequences related to the affected vessels [9]. The concept of an antiphospholipid inner ear syndrome has been recently proposed, given the elevated levels of anticardiolipin and anti-B2 glycoprotein antibodies and the presence of lupus anticoagulant found in 25% of patients with idiopathic progressive SNHL [9].

Although with conflicting evidence regarding its utility, anti-HSP70 antibodies have been the most frequently described serologic marker in AIED. Animal and human studies have shown an increased expression of anti-HSP70 in patients with AIED [4,14], but increased levels of anti-HSP70 have shown to have low sensitivity and specificity for AIED [4] whilst elevated titers of these antibodies failed to alter cochlear function in mice [15]. In the cohort presented, anti-HSP70 antibodies were also not relevant to predict an associated SIAD. Interestingly, ANAs were significantly more frequent in these patients. ANAs are a diverse group of autoantibodies that target components of the cell nucleus [16] and are important in the evaluation of patients with autoimmune diseases. ANA positivity can be present in healthy individuals and most people with ANA will probably never develop an autoimmune disease [16]. However, in specific populations such as patients with SNHL, ANA positivity might justify the decision for further diagnostic investigation regarding the presence of an associated SIAD. As in other organ-specific autoimmune diseases such as Hashimoto’s thyroiditis or autoimmune gastritis, AIED might be more frequent in patients with SAID but the association does not seem to affect the overall prognosis. Nevertheless, these patients have more prolonged follow-ups and are more frequently treated with non-steroid immunosuppressant drugs.

The majority of the available evidence defines prognosis solely on the basis of the response to treatment with steroid or other immunomodulatory drugs [1,17,18]. This response is usually defined as a non-standardized mean pure-tone average or word intelligibility score improvement from baseline. In some cases, these data are interpreted regardless of the severity of the hearing loss maintained at the last audiometric evaluation [1,17,18]. In this cohort, the authors choose to evaluate prognosis based solely on the results of the last pure tone audiogram available, with a normal audiometry and a mild hearing loss being considered an indicator of good prognosis and being present in one-third of the ears evaluated. Three reasons were considered for such prognosis definition: the global prognosis of affected ears/patients and not the steroid response was the purpose of the evaluation; it was imperative to find a definition of prognosis that was independent of the basal hearing loss in order to understand how initial disease severity would impact the outcome; lastly, and probably most important, the authors found that mild improvements should be considered an insufficient response if follow-up audiograms showed a significant remaining hearing loss, resulting in relevant morbidity to the patient. In the multivariate analysis a normal audiogram or a mild hearing loss and a bilateral involvement at the time of presentation were the only characteristics independently associated with prognosis: a mild disease at baseline was associated with good prognosis whilst bilaterality was associated with a poor one. Of note, mild disease at presentation could represent early diagnosis with the consequent early treatment, but it should be taken into account that neither time from first symptoms to diagnosis nor systemic or transtympanic infusion of corticosteroids seem to have influenced prognosis. Therefore, a mild disease at the time of presentation could eventually translate into a less progressive disease and correlate with a better prognosis. 

Of all the considered treatment options (transtympanic corticosteroids, systemic corticosteroids, or non-steroid immunomodulators), none seemed to have influenced prognosis. In 1997 Rauch et al. [19] published the empirical systemic corticosteroid treatment protocol of the Massachusetts Eye and Ear Infirmary. Adult patients would receive 60mg of prednisone daily for four weeks and would be considered steroid responders if their hearing threshold improved by 15 dB or more at one frequency and 10dB at two or more consecutive frequencies. Rapid tapering would then be offered to non-responders with responders receiving full-dose therapy until a plateau of recovering was achieved, followed by a maintenance dose for a variable length of time [19]. Transtympanic corticosteroid therapy has been used to deliver higher intra-labyrinthine concentrations whilst eliminating systemic side effects [13]. Despite the limited evidence supporting this approach, it has been used in patients refractory or intolerant to systemic steroids [13]. Although corticosteroids remain the mainstay of AIED treatment [6,7] and an initial response is reported in up to 70% of patients [6,9,13], some authors have stated that only 14% of patients remain steroid-responsive after 34 months of treatment [20]. This study did not evaluate a specific treatment protocol. However, as cumulative corticosteroid doses did not vary between patients with different prognosis, and given that the mean follow-up time of the cohort was 29 months, it is possible that patients could have entered a “steroid non-responder phase”. Other immunomodulatory drugs were used, but mostly in patients with associated SAID and did not change the outcome. One patient with SLE was treated with rituximab and maintained a bilateral profound hearing loss in the last audiometric evaluation.

As a retrospective analysis, this study has many limitations. It did not follow a specific treatment and diagnostic protocol and last pure tone audiograms were performed at different disease stages. Speech discrimination testing and vestibular symptoms and tinnitus were not used to define prognosis. Finally, hearing threshold fluctuations, frequent in AIED, may have also influenced the overall prognosis.

## Conclusions

In this cohort, SAID was present in one-third of the patients with suspected AIED. A normal audiometry or mild disease at presentation was associated with a good outcome, whilst bilateral involvement was associated with a bad one. Association of a SAID did not seem to influence the hearing-related prognosis. Determination of ANA may have a role in patients with suspected AIED to justify performing a complementary investigation to determine the presence of a SAID. It is important to achieve a future consensus on the definition of hearing improvement that considers the morbidity caused by remaining hearing loss, even if some hearing recovery is achieved with therapy.
